# PSYCHOSOCIAL PREDICTORS OF THE GENDER DIFFERENCE IN DEPRESSION OF OLDER ADULTS IN THE ORANJ BOWL PANEL

**DOI:** 10.1093/geroni/igz038.3162

**Published:** 2019-11-08

**Authors:** Christine V Ferri, Kaite Yang, Joan Girgus

**Affiliations:** 1 Stockton University, Atlantic City, New Jersey, United States; 2 Stockton University, Galloway, New Jersey, United States; 3 Princeton University, Princeton, New Jersey, United States

## Abstract

Recent reviews show that the gender difference in depressive symptoms that emerges in adolescence persists in older adults (Girgus, Yang & Ferri, 2017; Salk, Hyde & Abramson, 2017). However, researchers have yet to explain why this gender difference occurs in older adults and if it is for the same reasons as in younger ages. The present study used data collected from 3008 participants over the age of 60 (M=66.45, SD=4.36) from the baseline wave of the ORANJ BOWL, a longitudinal study based in New Jersey (Pruchno, Wilson-Genderson, Rose, & Cartwright, 2010). Depressive symptoms were measured with the CES-D. Six psychosocial predictors of the gender difference in depression were analyzed: functional ability, social support, perceived financial comfort, income level, marital status, and living alone. Women reported significantly more depressive symptoms compared to men (p<.001). Women had significantly lower functional ability, physical health, financial comfort, income level, but more social support than men (all p’s<.02). Women were more likely to live alone and to be separated, divorced, or widowed (all p’s<.001). Significant interactions for gender x functional ability, gender x social support, and gender x living alone were found in predicting depression using hierarchical linear regression. Living alone and poor function were more detrimental for men than women. Low social support was more detrimental for women than men. Understanding the gender difference in depressive symptoms will allow for targeted screening and preventative interventions for women and men at highest risk for depression.

